# Spray-coated perovskite hemispherical photodetector featuring narrow-band and wide-angle imaging

**DOI:** 10.1038/s41467-022-33934-1

**Published:** 2022-10-15

**Authors:** Xiaopeng Feng, Yuhong He, Wei Qu, Jinmei Song, Wanting Pan, Mingrui Tan, Bai Yang, Haotong Wei

**Affiliations:** 1 State Key Laboratory of Supramolecular Structure and Materials, College of Chemistry, Jilin University, Changchun, 130012 China; 2 Optical Functional Theragnostic Joint Laboratory of Medicine and Chemistry, The First Hospital of Jilin University, Changchun, 130012 China

**Keywords:** Electronic devices, Electronic materials, Optical sensors, Optomechanics

## Abstract

Sphere imagers featuring specific wavelength recognition and wide-angle imaging are required to meet the fast development of modern technology. However, it is still challenging to deposit high-quality photosensitive layers on sphere substrates from low-cost solution processes. Here we report spray-coated quasi-two-dimensional phenylethylammonium/formamidinium lead halide (PEA_2_FA_n-1_Pb_n_X_3n+1_) perovskite hemispherical photodetectors. The crystallization speed is manipulated by perovskite compositions, and the film thickness can be controlled by spray-coating cycles and solution concentration from tens of nanometers to hundreds of micrometers with a fast velocity of 1.28 × 10^−4^ cm^3^ s^−1^. The lens-free hemispherical photodetectors allow light response at a wide incident angle of 180°. Simultaneously, the wavelength selective response from visible to the near-infrared range is achieved with full width at half maximums (FWHMs) of ~20 nm, comparable to single-crystal devices. Wide-angle and wavelength-selective imaging are also demonstrated, which can find potential applications in intelligent recognition and intraoperative navigated surgery.

## Abstract (web summary)

Hemispherical photodetectors allow wide sight angle without the complex optical paths of fisheye lenses. Here, Wei et al, report a spray-coated quasi-two-dimensional perovskite hemispherical photodetector with wavelength selective response from the visible to the near-infrared.

## Introduction

Advanced photodetectors that can sense specific light wavelengths have been widely deployed in many fields, such as face recognition, driverless navigation, intraoperative navigated surgery, surveillance systems, and robotics for inspection and rescue^1–7^. However, planar sensors have their intrinsic limitation in broadening the sight angle for more information, and only a 60° sight angle is expected for standard planar devices^8^. Although the fisheye lens can improve this value to over 180°, the complex optical path will also add more cost to the integrated wide-angle sensor, which is often fragile, instability of collimation, and limited in focal length^9^. Inspired by the fisheye and compound-eye architecture of arthropods, a hemispherical photosensor^10–12^ can provide a wide enough sight angle to solve this problem. While depositing insoluble semiconductors such as Si and InGaAs onto the substrate through gas vapor deposition suffers high cost and low deposition speed^13,14^. In addition, specific recognition is another essential figure-of-merit for a modern photodetector to meet the fast-increasing demands of technology. Depositing a soluble active layer to fabricate photosensors on required non-planar substrates to respond to specific wavelength light at a wide angle is still challenging^12,15^.

Spray-coating is a mature technology that has been widely employed for car spray painting and ferry anticorrosion coating^16,17^. The low-cost solution processes can deposit layered thin films on desired substrates of irregular shape^18–20^. In contrast to the spin-coating or doctor-blading technique, the spray-coating technique is not limited by the shape of the substrate. In addition, spray-coating is a pressure-driven coating process, and sprayed solvents often volatilize very fast under strong airflow before depositing on substrates. Therefore, the subsequent layer doesn’t dissolve the previous layers since the deposited solution is already supersaturated, and the thickness of the deposited films can be uniformly scaled up to hundreds of micrometers or even millimeters^21^.

Quasi-two-dimensional (quasi-2D) perovskites are solution-processable and combine the advantages of excellent charges carriers’ dynamics behavior of 3D perovskite and good stability of 2D perovskite^22–26^. Their unique optoelectronic properties of anisotropic quantum confinement, tunable bandgap, and defect tolerance nature enable attracting applications in photovoltaic^27,28^, light-emitting diode (LED)^29,30^, laser^31^, X-ray detectors^32–35^, and photodetectors^4^. Spray-coated perovskite films for solar cells have been reported for large area devices^36,37^. Nevertheless, the most prominent advantage of the technique lies in the uniform deposition of active layers on irregularly shaped substrates, which has never been carefully studied. In recent days, sphere perovskite LED has been reported based on a close-spaced vapor reaction (CSVR) process, filling the gap in irregularly shaped perovskite LED devices^38^.

In this article, we report the spray-coated quasi-2D phenylethylammonium/formamidinium lead halide (PEA_2_FA_n-1_Pb_n_X_3n+1_) perovskite hemispherical photodetectors that can collect images upon specific light wavelength at a wide light incident angle of 180°. The crystallization process of the spray-coated quasi-2D perovskite film is manipulated. The narrow-band response is realized by controlling the charges carriers’ dynamic behavior in a thick hemispherical perovskite device, and wide-angle imaging is also demonstrated.

## Results

### Spray-coated hemispherical perovskite films

**Fig. 1:**
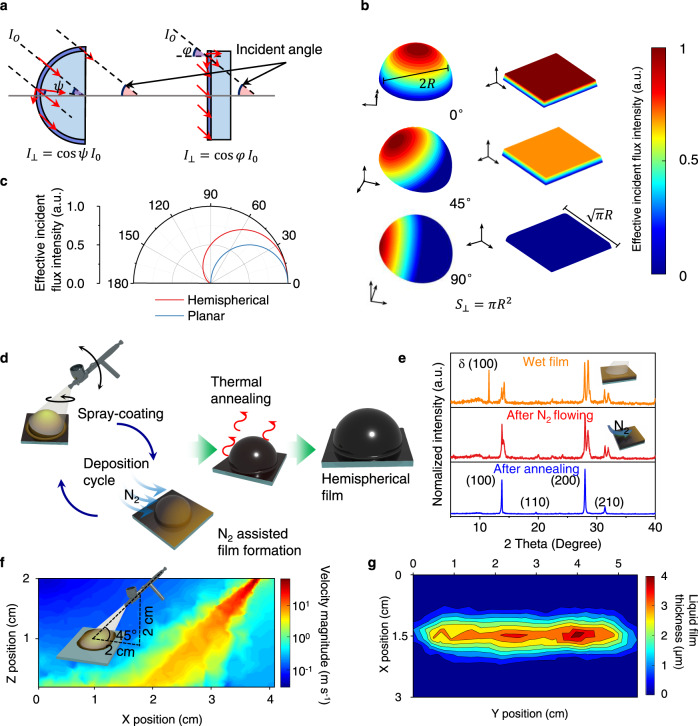
The effective incident flux intensity on hemispherical and planar surface, and thick perovskite films by spray-coating. **a** Schematic diagram of the vertical component of incident light on hemispherical and planar surface. The incident light (red arrow) is decomposed into components parallel and perpendicular to the surface. *ψ* and *φ* are angles to evaluate the angle of incident light. **b** The simulated spatial distribution of effective incident flux incident of planar and hemispherical surface (${S}_{\perp }=\pi {R}^{2}$) under incident light from 0°, 45°, and 90°. **c** The integrated ${I}_{\perp }$ flux under incident light from incident light from different angles. **d** Schematic diagram of the film fabrication process through spray-coating. **e** The XRD spectra of the perovskite film in each step of spray-coating process. **f** The simulation of fluid field’s velocity magnitude distribution (The angle of spray-coating is 45°) **g** The simulation of liquid film thickness (linear dynamic spraying: 5 cm s^−1^) at the first second.

Information capture is one of the most important aspects of modern detection science. The absorption of incident light is essential for accurate and sensitive photodetection. Therefore, detecting light at a wide-angle is important for photodetection. However, the effective incident flux intensity of a planar device can decrease sharply as the increase of incident light angle. The effective incident flux intensity at the hemispherical and planar surfaces is discussed. We assume that the area of incident light is larger than the size of photodetectors. *I*_⊥_ is the vertical component of incident light (*I*_*O*_) after vector decomposition. Psi (*ψ*) and phi (*φ*) are angles to evaluate the incident light directions at hemispherical and planar surfaces, respectively, as shown in Fig. 1a. Figure 1b shows our *I*_⊥_ simulation results of the effective incident flux intensity at the hemispherical and planar surfaces with the light incident angle of 0°, 45°, and 90°, respectively. It can be clearly seen that the ${I}_{\perp }$ largely decreases at 45° and reaches zero at 90° light incident angle, which is much smaller than that at the hemispherical surface. The integrated ${I}_{\perp }$ flux from different incident angles is calculated in Fig. 1c, and the incident flux at the hemispherical surface is always higher than the planar one. Therefore, the hemispherical photodetectors can collect more information regarding different incident directions of light, which is more convenient for wider vision or object positioning.

The scheme of the spray-coating processes is illustrated in Fig. 1d, and we rotate the substrate during the spray-coating for uniform film deposition. Hemispherical substrates with cleaned surfaces are put on a hot plate before spraying. After each spraying pulse onto substrates, high speed nitrogen (N_2_) gas is employed to treat the substrate surfaces and accelerate films crystallization^24,39–41^. Finally, the films are thermally annealed to further improve crystallization quality. The surfaces of the films are polished to obtain a smooth and compact film with fewer surface defects (Supplementary Fig. 1). The film formation and crystallization processes during spray-coating are characterized by powder X-ray diffraction (XRD) in Fig. 1e. The just spray-coated wet film shows a clear yellow phase of FAPbI_3_ perovskite, and N_2_ can assist the formation of black phase. Further annealing process promotes the fast crystal growth and exhibits narrow XRD peak. The pneumatic spray method is facile for rapid film preparation. To better understand spray-coating process, the computational fluid dynamics (CFD) method is applied based on the principle of fluid mechanics. We employ the standard *k*-*ε* turbulence model to describe the spray process, which is governed by a two-phase flow^16,42^. The gas inlet is positioned at 45° from the horizontal plane with a height of 2 cm from the substrate. The flow velocity distribution of the precursor is simulated in Fig. 1f, and corresponding liquid film thickness distribution is described in Fig. 1g. The linear dynamic spray-coating is executed where the source is moved parallel to the y-axis at a constant velocity of 0.50 cm s^−1^. The precursor can be uniformly deposited on the substrates for a large distance. To get uniform films on a two-dimensional surface, the moving profile of the spray gun should keep a spacing distance of ~0.8 cm as shown in Supplementary Fig. 2^17^.

The spray-coated solid perovskite film thickness (*H*_*s*_) can be calculated by the following Eq. (1),

1 $${H}_{s}={H}_{l}\frac{{V}_{s}}{{V}_{l}} k$$

where *H*_*l*_ is the thickness of sprayed liquid film, *V*_*l*_ is the volume of liquid film, *V*_*s*_ is the volume of the solid film, and *k* is the usage efficiency of the precursor solution. Since sprayed droplets will stick together by forming a thin layer of the liquid film. The velocity of thin film deposition by spray-coating can reach 1.28 × 10^−4^ cm^3^ s^−1^ (Supplementary Fig. 2). To verify the velocity, 2.0 mL quasi-2D PEA_2_FA_3_Pb4I_13_ perovskite in mixture solution (N, N-Dimethylformamide (DMF)/Acetonitrile (ACN) = 1:1, v/v) with concentration of 0.6 M was spray-coated on a 36 cm^2^ substrate. It takes us only 15 min to obtain a uniform film with thickness of ~28 μm (Supplementary Fig. 3b), in consistent with the 32 μm of simulation result in Supplementary Fig. 2. The thickness of the different deposition times was collected and shown in Supplementary Fig. 3a. Therefore, the thickness of the film is controllable by the spray-coating time and cycles.

**Fig. 2:**
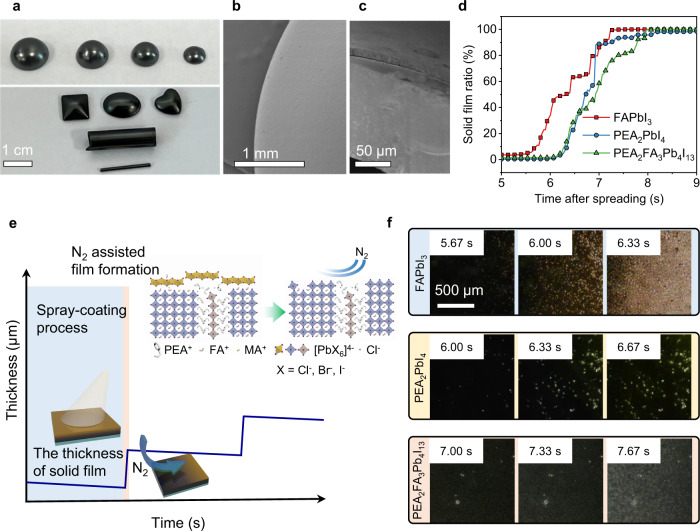
The thick perovskite film fabricated by spray-coating and crystallization process. **a** The optical photographs of spray-coated perovskite (PEA_2_FA_3_Pb_4_I_13_) films deposited onto different substrates of different sizes and shapes. **b** The SEM image of the surface of the perovskite film on hemispherical substrate. **c** The cross-section SEM image of the perovskite film on curved substrate. **d** The time history of solid film formation ratio (the crystallization processes of different perovskites). **e** Schematic diagram of the solid film thickness during spray-coating and N_2_ flowing process. **f** The microscope images of the crystallization processes of different perovskites precursors drop-coated on the hot plate.

Figure 2a shows the photos of spray-coated PEA_2_FA_3_Pb_4_I_13_ perovskite films on several substrates with different shapes and sizes. Large-area and uniform perovskite films can be facilely deposited through spray-coating. The surface and cross profile morphology images were acquired by scanning electron microscope (SEM), as shown in Fig. 2b, c, exhibiting uniform and compact films. The thickness of the spray-coated film can be gradiently manipulated by controlling the spraying cycles in Fig. 2e. The liquid perovskite solution is sprayed under high-pressure spray gun, and the sprayed solvent easily volatilizes during spraying process. Therefore, the subsequently deposited perovskite solution is often supersaturated and does no’t dissolve the perovskite films on substrates.

### Crystallization management and charge carriers’ dynamic behavior

To study the crystallization processes of spray-coated perovskite films, we employed a microscope to record the film formation of 3D FAPbI_3_, 2D PEA_2_PbI_4_, quasi-2D PEA_2_FA_3_Pb_4_I_13_ perovskites in Fig. 2f to study the nucleation and crystal growth processes. The entire crystallization process only takes a few seconds, and we observed that the crystallization of 3D FAPbI_3_ is earlier than 2D PEA_2_PbI_4_. The velocity difference should be caused by the solubility difference as illustrated in Supplementary Fig. 4. It should be noted that the crystallization velocity of quasi-2D PEA_2_FA_3_Pb_4_I_13_ is the slowest, which may result from the interactions between 3D and 2D perovskites. According to the video of film formation processes, we summarize the film formation ratios at different time in Fig. 2d. Two-dimensional perovskite has a sudden turning point at the 7^th^ second for crystallization, and similar case occurs at the 7.8^th^ second in quasi-2D perovskite. Therefore, partial phase separation exists at this point, and the 2D layers should cap the crystallized 3D grains^43^. Nitrogen gas promotes the evaporation of the solvent and the nucleation of perovskite at the gas-liquid interface, with (MA-FA)PbI_3_ precipitating. MA comes from the additive of methylammonium chloride (MACl), which is widely used in FA-based perovskites for forming and stabilizing the black phase at a lower temperature^28^. A small amount of δ–phase FAPbI_3_ was observed from XRD study, which can be further removed by following thermal annealing^40,44^. The crystallization is completed in the end with MACl removed at high temperature.

**Fig. 3:**
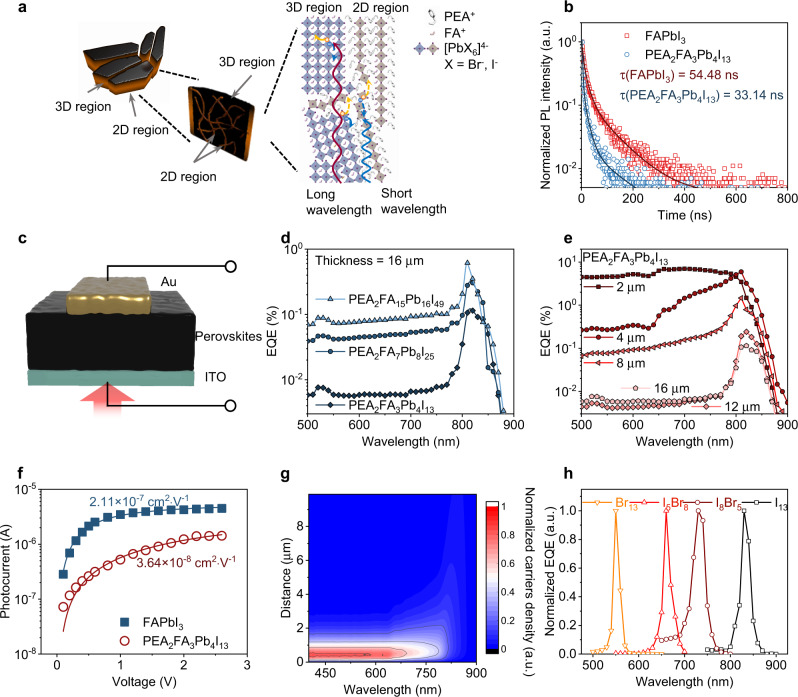
The charge carriers’ transport in spray-coated thick film and narrow-band photodetector. **a** Schematic diagram of the perovskites (PEA_2_FA_n-1_Pb_n_X_3n+1_) films fabricated by spray-coating. The recombination occurs at the grain boundary (2D region), selectively collecting long wavelength-generated carriers and narrow-band response. **b** The TRPL spectra of PEA_2_FA_3_Pb_4_I_13_ (*τ* = 33.14 ns) and FAPbI_3_ (*τ* = 54.48 ns) fabricated by spray-coating. **c** The device structure to analyze the influent of narrow-band response photosensitive layer. **d** The EQE value of devices (film thickness: 16 μm) based on perovskites with different *n* value. **e** The EQE value of devices based on PEA_2_FA_3_Pb_4_I_13_ films of different thickness (2 μm, 4 μm, 8 μm, 12 μm, and 16 μm). **f** The μτ product of PEA_2_FA_3_Pb_4_I_13_ and FAPbI_3_ films fabricated by spray-coating. **g** The charges density of PEA_2_FA_3_Pb_4_I_13_ film at different wavelength and positions simulated by diffusion length and absorbance spectrum. **h** The narrow-band response (EQE) of perovskites photodetectors with different halogen ratio (PEA_2_FA_3_Pb_4_I_13_, PEA_2_FA_3_Pb_4_I_8_Br_5_, PEA_2_FA_3_Pb_4_I_5_Br_8_, and PEA_2_FA_3_Pb_4_Br_13_).

The film XRD spectra confirm that the 2D perovskite (PEA_2_PbI_4_) exists in the grain boundary. The XRD peaks identification refers to only 3D FAPbI_3_ perovskites (Supplementary Fig. 5a). The transient absorption (TA) spectra also confirm the two species in the film fabricated by spray-coating, and no alloying is observed^22^. Through studying the ultraviolet photoelectron spectra (UPS) data, the surface work function of PEA_2_FA_3_Pb_4_I_13_ fabricated by spray-coating is close to the FAPbI_3_ (Supplementary Fig. 6a), consistent with the absorbance spectra and photoluminescence (PL) spectra (Supplementary Fig. 6b) results. One figure-of-the-merit of an advanced photodetector is the filterless narrow-band response^2,15,45–47^. To resolve the selective wavelength light, it is essential to tune the charges carriers’ dynamic behavior of photosensitive layers. Since 2D perovskites are uniformly distributed around 3D perovskite grains, the charge carriers’ dynamic behavior can be tuned by the ratio of 2D/3D perovskites. To confirm this, the time-resolved photoluminescence spectra (TRPL) are performed in Fig. 3b, which show a large lifetime difference between quasi-2D (33.14 ns) and pure FAPbI_3_ (54.48 ns) due to the high exciton binding energy of 2D perovskite^48,49^. We fabricate several devices with the structure of ITO/PEA_2_FA_n-1_Pb_n_I_3n+1_/Au in Fig. 3c. We change the ratio of 2D/3D perovskites by employing quasi-2D perovskites with n values equaling 4, 8, and 16, and corresponding external quantum efficiency (EQE) spectra of quasi-2D perovskite devices are presented in Fig. 3d. PEA_2_FA_3_Pb_4_I_13_ perovskite with a low n value of 4 leads to a high cut-off ratio (EQE_820 nm_/EQE_700 nm_) of around 17 (Supplementary Fig. 7e), indicating more 2D composition ratio can effectively induce more charges carrier recombination upon short wavelength excitation. Corresponding full width at half maximums (FWHMs) is around 20 nm. Furthermore, we tuned the thickness of the perovskite layer to match the light penetration depth and charge carriers’ diffusion length. The narrow-band response gradually shows up as the thickness of the spray-coated perovskite film increases from 2 µm to 16 µm. The FWHM can be smaller than 20 nm with a cut-off ratio of 42 (Supplementary Fig. 7f), which is already comparable to single-crystal devices. Mu-tau (*µτ*) product is an important parameter that is proportional to the charges carriers’ diffusion length (*L*_*D*_). The measured *µτ* product of the spray-coated FAPbI_3_ and PEA_2_FA_3_Pb_4_I_13_ films by photoconductivity methods are 2.11 × 10^−7^ cm^2^ V^−1^ and 3.64 × 10^−8^ cm^2^ V^−1^, respectively (Fig. 3f). The electrons mobility was measured in the PEA_2_FA_3_Pb_4_I_13_ film of 6.52 × 10^−5^ cm^2^ V^−1^ s^−1^ (Supplementary Fig. 7d). The *L*_*D*_ describes the diffusion capacity of non-equilibrium carriers which can be calculated by the following Eq. (2)^50^,

2 $${L}_{D}=\sqrt{\mu \tau \frac{{k}_{B}T}{q}}$$

where *k*_*B*_ is Boltzmann constant, *q* is the absolute value of electron charge, and *T* is temperature. Based on the steady-state one-dimensional diffusion model, the charge density can be described as following Eq. (3)^51,52^,

3 $$n\left(x\right)=\frac{g{N}_{0}}{\delta }\frac{\alpha {{L}_{D}}^{2}}{1-{\left(\alpha {L}_{D}\right)}^{2}}({e}^{-\alpha x}-{e}^{-\frac{x}{{L}_{D}}})$$

where *n* (*x*) is the charges density at *x* position, *g* is the internal efficiency of photon-to-charge, *N*_*0*_ is the number of incident photons, *α* is the absorption coefficient of the front layer material, and *δ* is the charge diffusion coefficient. Since charge carriers will gradually recombine during diffusion, this equation can reflect the relationship between film thickness and charges collection efficiency. The distribution of charges (Fig. 3g, Supplementary Fig. 7b, c) is simulated by considering the absorbance coefficients and the charge carriers’ diffusion length. Short-wavelength light generates charges at the top surface, which results in a fast signal loss by charges recombination. In contrast, long-wavelength light has a larger penetration depth and narrow-band responsivity. To tailor the wavelength response range, PEA_2_FA_3_Pb_4_X_13_ perovskites with different I/Br ratios are adopted. The devices are constructed as ITO/PEDOT:PSS/PEA_2_FA_3_Pb_4_X_13_/C_60_/BCP/Au, as shown in Supplementary Fig. 8a, and the buffer layers are inserted to suppress the device leakage current and maintain the narrow-band response. The EQE spectra can be continuously tuned from visible light to near-infrared range as shown in Fig. 3h, and the FWHM is around 26 nm with a high cut-off ratio of 20 upon −0.6 V applied bias (Supplementary Fig. 8b). The responsivity and specific detectivity at the response wavelength of this device are 35 mA W^−1^ and ~10^11^ Jones, respectively (Supplementary Fig. 8c, f).

### Hemispherical device for narrow-band response and wide-angle imaging

**Fig. 4:**
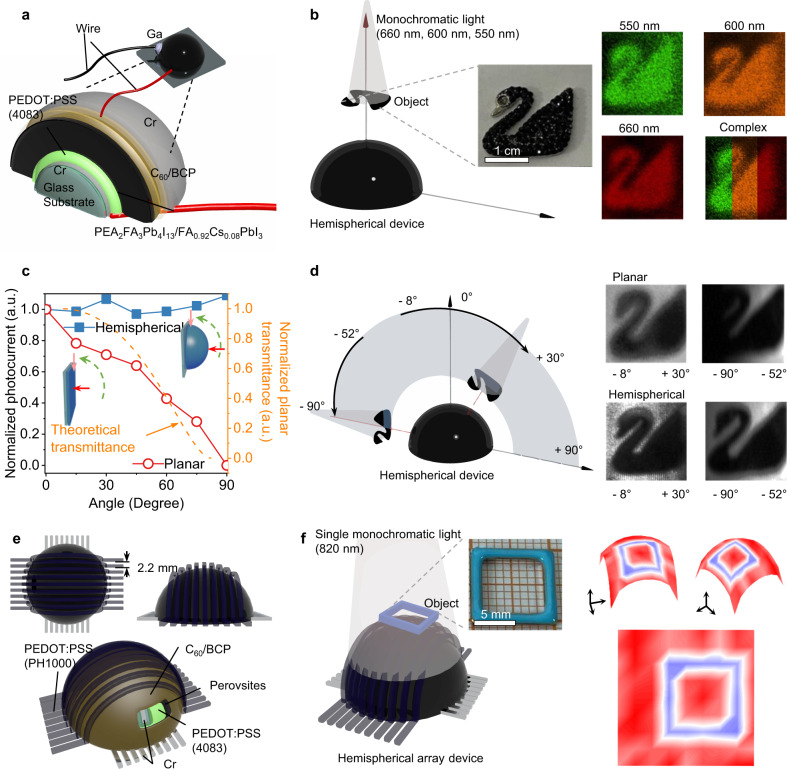
Narrow-band and Wide-angle imaging of a hemispherical photodetector. **a** The device structure of the hemispherical photodetector. **b** Left: The schematic of the imaging system and the optical photo of a swan as object. Right: The images captured by hemispherical photodetectors with different I/Br ratios. The colors of images are matched to the color function. **c** The photocurrent of hemispherical photodetector under irradiation from different angles (The size of the light source is smaller than the area of photodetectors.). The orange line is the relationship between the theoretical normalized transmittance and the incident angle of a planar perovskite film surface. **d** Left: The schematic of the imaging system for wide angle detection. Right: The images captured by planar and hemispherical photodetectors based on different angles of incident light. **e** The schematic of the structure of the micro-array (9 × 9) device **f** Left: The schematic of the imaging system and the optical photo of a square plastic object. Right: The micro-array imaging result.

To construct a hemispherical device, chromium (Cr) as bottom electrodes are deposited on the commercial hemispherical glass substrates to improve the substrate adhesion. The diameters of the hemispherical glass substrates are 0.8 cm. Buffer layers and PEA_2_FA_3_Pb_4_I_13_ perovskites are subsequently deposited, as illustrated in Fig. 4a, and 8 nm Cr is deposited as semi-transparent top electrodes. PEDOT:PSS is the hole transport layer with a thickness of ~125 nm measured by the surface profiler. The thickness of the quasi-2D perovskite layer and the 3D perovskite buffer layer is about 10–16 μm and 3–5 μm, respectively. The SEM images of the surface of the hemispherical photodetector were studied in Supplementary Fig. 9a and  9b. C_60_ is the electron transport layer with a thickness of around 30 nm deposited onto the surface of perovskites. BCP is the buffer layer with a thickness of about 8 nm. We also fabricated n-i-p photodetectors with the device structure of Cr/SnO_2_/perovskite/PTAA/Cr. SnO_2_ is the electron transport layer with a thickness of about 150 nm, and PTAA is the hole transport layer with a thickness of around 80 nm. The current density (J)-voltage (V) curves at different light flux intensities are shown in Supplementary Fig. 9c. The dark current density is 3.34 × 10^−8^ A cm^−2^ at −0.4 V bias condition, which is much lower than other perovskites-based photodiodes due to the compact and thick perovskite layer and energy barrier constructed by buffer layers. The photocurrent density is 1.34×10^−6^ A cm^−2^ upon 97 µW cm^−2^ light irradiation. The responsivity and specific detectivity at the response wavelength obtained from J-V curve are 13.8 mA W^−1^ and ~10^11^ Jones, respectively. Hemispherical photodetectors with selective wavelength responses at 550 nm, 600 nm, and 660 nm are also fabricated by tuning the halide I/Br ratios. Supplementary Fig. 9e shows the relationship between the response wavelength and I/Br ratio. The normalized EQE spectra of narrow-band hemispherical photodetector are shown in Supplementary Fig. 9d. We noticed that the cut-off ratio of hemispherical device is lower than the planar device, which may be caused by the low conductivity of bottom Cr electrodes. Based on the narrow-band response of hemispherical devices, we set up a homemade imaging system by recording the photocurrent signal with a lock-in amplifier, and the monochromatic LED is modulated by a function generator in Fig. 4b. Two-dimensional X-Y stage is programmatically controlled with a step distance of 500 µm. The relative position details of light source, object, and photodetectors were shown in Supplementary Fig. 10b, c. The right panel of Fig. 4b shows the swan images captured by hemispherical detectors with a specific light response to 550 nm, 600 nm, and 660 nm. The colors of images correspond to the RGB coordinates by considering the EQE spectra, and composite image is acquired by compositing the images, realizing a specific spectral recognition and color imaging.

To explore the wide-angle detection performance of hemispherical devices, the photocurrent of the hemispherical device versus incident light angle is recorded in Fig. 4c, and the photocurrent of planar device is also examined under the same conditions. The hemispherical device shows independent photocurrent upon different light incident angles from 0° to 90°. However, the photocurrent of planar device linearly decreases as the incident angle due to the limited light absorption at inclined directions. No signal can be observed when the incident light is parallel to the planar device. We also demonstrate the wide-angle imaging capability of the hemispherical device in Fig. 4d. The swan as an object is placed at the sight angle range between −90° and −52°, and clear swan image is recorded in the right panel of Fig. 4d. The image is the same with the sight angle from −8° to 30°, showing a spatial resolution of 1.78 lp mm^−1^ (Supplementary Fig. 11)^53^. In contrast, the planar device totally loses signal at a −90° sight angle, and only half image of swan is acquired under same condition, highlighting the advantages of hemispherical photodetector.

To realize imaging under single light illumination like natural daylight or moon light, preliminary micro-array imaging was also demonstrated by array pixels on a hemispherical substrate. The device structure of each single pixel is Cr/PEDOT:PSS (4083)/Perovskite/C_60_/BCP/PEDOT:PSS (PH1000) as shown in Fig. 4e. The diameter of hemispherical photodetector is ~3 cm. Bottom electrode is Cr, which is deposited by vacuum evaporation through a 3D printed mask. Surface electrode is PEDOT:PSS (PH1000), which is deposited by spray-coating covered by a mask to form intersecting pixels. In the preliminary demonstration, a photodetector arrays with 9 × 9 pixels (1.21 mm^2^) were fabricated, and the distance between the two electrodes is 2.2 mm. The schematic of the imaging system is shown in Fig. 4f, where a square plastic subject was placed between the light source and detector. More details were shown in the Supplementary Information. The smoothed array imaging result was recovered to the hemispherical surface and is shown in Fig. 4f, showing no theoretical limitation in large area micro-array imaging.

## Discussion

In summary, we report a perovskite hemispherical photodetector through fast spray-coating processes, which performs lens-free imaging capability at a wide-angle range of nearly 180°, greatly reducing the dependency on complex optical components. The spray-coated perovskite films are large-area, uniform, compact, and compatible to irregularly shaped substrates. The thickness of the perovskite layers can be easily adjusted by controlling the spray-coating cycles. Through manipulating the charges carriers’ dynamic behavior in different quasi-2D perovskite compositions and film thicknesses, narrow-band response from visible to near-infrared range is realized on hemispherical perovskite device with FWHMs smaller than 20 nm. The responsivity and specific detectivity at the response wavelength of the hemispherical photodetector is 13.8 mA W^−1^ and ~10^11^ Jones, respectively. This work also provides a path to fast films deposition and stereoscopic films formation for advanced optoelectronic semiconductor devices with large areas and irregular shapes.

## Methods

### Photodetectors fabrication

Twelve nanometers of Cr were evaporated onto the hemispherical substrate (glass). Substrates (ITO or Cr coated hemispherical substrate) were treated by UV-O_3_ for 20 min before plasma treatment of 5 min. The area for spray-coating is 25 cm^2^. The PEDOT:PSS (4083) solution (~1%_wt_ aq.) is diluted by EtOH (stock solution:EtOH = 1:19, v/v). The volume of the precursor is 800 µL. The spray-coating equipment was composed of an air compressor and gravity spray gun with a jet nozzle of 0.2 mm. Substrates were fixed on a thin metal plate and heated to 75 °C before the first layer of the precursor was spray-coated onto them. Then dry N_2_ blew onto films through a pneumatic gun. Until most of the solvent was removed, the next layer was precipitated onto the former layer. After spray-coating, the film is annealed under 100 °C for 10 min. The thickness of the PEDOT:PSS layer is about 125 nm. PEA_2_FA_3_PbI_x_Br_13-x_ (0.6 M, x = 0, 2, 5, 8, and 13) was dissolved in DMF/ACN mixed solvent (1: 1, v/v) as the precursor. For example, FAI (92.9 mg), PEAI (89.6 mg), PbI_2_ (332 mg), MACl (14.5 mg, 30%_mol_ equivalent to Pb^2+^), L-Ascorbic Acid (L-AA)^54^ (6.34 mg, 5%_mol_ equivalent to Pb^2+^) were dissolved in 1.2 ml DMF/ACN mixed solvent. Substrates were heated to 100 °C. When it comes to spherical substrates, the glass plate needs to be rotated continuously. Films after spray-coating were annealed at 120 °C for 30 min to remove the solvent and crystallization preliminarily. Then films were heated to 170 °C for 60 min to form the FA^+^ based perovskites of α-phase finally. FA_0.92_Cs_0.08_PbI_y_Br_3-y_ (0.4 M, y = 3, 1.85, 1.15, 0.46, 0) was dissolved in DMF/2-Me mixed solvent (1: 1, v/v) as the precursor. For example, FAI (50.6 mg), CsI (6.65 mg), PbI_2_ (148 mg), MACl (6.4 mg, 30%_mol_ equivalent to Pb^2+^), L-AA (2.8 mg, 5%mol equivalent to Pb^2+^) were dissolved in 0.8 ml DMF/2-Me mixed solvent. The perovskite precursor was spray-coated onto the surface of the PEA_2_FA_3_PbI_x_Br_13-x_ at the same condition as a buffer layer. After spray-coating, the surface of perovskites was polished by polishing paper and cotton. C_60_ (30 nm), and BCP (8 nm), were evaporated onto the surface of perovskites. Chromium electrodes (8 nm) were evaporated onto the devices. Wires were connected the electrodes of hemispherical photodetectors assisted by gallium (Ga).

The film deposition step of the fabrication of n-i-p devices is same. The SnO_2_ solution (15%_wt_ aq.) is diluted to 0.25%_wt_ by water. The area for spray-coating is 25 cm^2^. The volume of the precursor is 800 µL. The temperature of substrates for spray-coating is 75 °C. The thickness of SnO_2_ is 150 nm. After spray-coating, substrates covered by SnO_2_ were annealed for 30 min at 170 °C. Before depositing the perovskites layer, substrates covered by SnO_2_ should be treated by UV-O_3_. After depositing the perovskites layer, the PTAA solution (1 mg cm^−1^ in toluene) was prepared. The volume of the precursor is 800 uL. The temperature of substrates for spray-coating is 75 °C. The thickness of PTAA is 80 nm. Before covering electrodes, films were annealed for 10 min at 100 °C.

The film deposition step of the fabrication of array devices is the same. The area for spray-coating is 25 cm^2^. The PEDOT:PSS (PH1000) solution (~1%_wt_ aq.) is diluted by EtOH (stock solution:EtOH = 1:19, v/v). The volume of the precursor is 1600 µL after spray-coating photodetectors were annealed for 15 min at 100 °C.

### *μτ* product and the simulation of carriers’ diffusion

The *μτ* product of perovskites was obtained by fitting the modified Hecht Eq. (4),

4 $$I=\frac{{I}_{0}\mu \tau V}{{L}^{2}}\frac{1-{e}^{-\frac{{L}^{2}}{\mu \tau V}}}{1+\frac{{Ls}}{V\mu }}$$

where *I* is the measured photocurrent, *I*_*0*_ is the saturated photocurrent, *L* is the thickness, and *V* is the applied bias. *s* is surface charge recombination rate. The absorption coefficient (α) was acquired by UV-Vis absorbance. The internal efficiency of photon-to-exciton is assumed as constant. For Eq. (3), $\frac{g{N}_{0}}{D}\frac{\alpha {L}^{2}}{1-{\left(\alpha L\right)}^{2}}$ is a constant (*k*_*n*_) independent on distance and wavelength. To describe the distribution of photon-generated carriers, Eq. (5) can be provided by

5 $$n\left(w,\, x\right)=k_{n}\left({e}^{-\alpha (w)x}-{e}^{-\frac{x}{L}}\right)$$

where *α* (*w*) is the absorption coefficient at the wavelength of *w*, *n* (*w*, *x*) is the charges density at the wavelength of *w*, and the position of *x*. At a certain wavelength, the total amounts of photon-generated carriers (*N*) is acquired by integrating Eq. (5),

6 $$N=\int {ndx}=k_{n}\left(-\frac{1}{\alpha }{e}^{-\alpha x}+L{e}^{-\frac{x}{L}}\right)+C$$

where *C* is arbitrary constant. The normalized distribution (*R* (*w, x*)) is demonstrated by Eq. (7).

7 $$R(w,x)=\frac{n(w,x)}{{N}_{0}^{{{{{{\rm{\infty }}}}}}}}$$

### The calculation of the distribution of ${I}_{\perp }$

For spherical objects, ${I}_{\perp }$ is constant with the angle changing of incident light. However, the distribution of ${I}_{\perp }$at the surface is different (Fig. 1a), which can be described by Eq. (8),

8 $${I}_{\perp }={{{{{{\rm{cos }}}}}}\psi I}_{O}$$

where *ψ* is the angle between the intersection of light rays with a sphere and light passing through the center of the sphere in the same direction. When it comes to planar objects, uniformly irradiated by light, ${I}_{\perp }$ is restricted by the angle of incident light, which can be described by Eq. (9),

9 $${I}_{\perp }={{{{{{\rm{cos }}}}}}\varphi I}_{O}$$

where *φ* is the angle between plane and light. The effective incident flux intensity $({\it\varPhi})$ of hemispherical surface can be described by following Eq. (10),

10 $$\it \varPhi={{{{{{\bf{I}}}}}}}_{{{{{{\bf{O}}}}}}}\cdot{{{{{\bf{S}}}}}}=\iint {{{{{{\rm{cos }}}}}}\psi I}_{O}{dS}$$

where *dS* the differential of area of hemispherical surface is expressed by the following function Eq. (11),

11 $${dS}={r}^{2}{{{{{\rm{sin }}}}}}\theta d\theta d\phi$$

where *r, θ, ϕ* are spherical coordinates. Thus, effective incident flux intensity is finally obtained in Eq. (12). When the incident angle is 0 degree, *θ* is equal to *ψ*.

12 $$\it {\varPhi}=\iint {I}_{O}{r}^{2}{{{{{\rm{sin }}}}}}\psi {{{{{\rm{cos }}}}}}\theta d\theta d\phi$$

## Supplementary information

Supplementary Information

## Data Availability

The data that support the plots within this paper are available from the corresponding author upon request. Source data are provided with this paper.

## Source data

Source Data
