# Supplementary material for: Spray-coated perovskite hemispherical photodetector featuring narrow-band and wide-angle imaging
Source: Nat Commun. 2022 Oct 15;13:6106. doi: 10.1038/s41467-022-33934-1 (PMC9569351; doi:10.1038/s41467-022-33934-1)
Supplement: Supplementary file 1 — Supplementary Information [file 41467_2022_33934_MOESM1_ESM.pdf]

# Supplementary Information for

# Spray-coated Perovskite Hemispherical Photodetector Featuring Narrow-band and Wide-angle Imaging

Xiaopeng Feng[1], Yuhong He[1], Wei Qu[1], Jinmei Song[1] ,Wanting Pan[1], Mingrui Tan[1], Bai Yang[1,2], and Haotong Wei[1,2*]

[1]*State Key Laboratory of Supramolecular Structure and Materials, College of Chemistry, Jilin University, Changchun, 130012, P. R. China*

[2]*Optical Functional Theragnostic Joint Laboratory of Medicine and Chemistry, The First Hospital of Jilin University, Changchun, 130012 P.R. China*

*Email: hweichem@jlu.edu.cn

**Supplementary Notes**

General information and materials ............................................................................3

**Supplementary Methods** ..........................................................................................4

**Supplementary Figures and Discussions** .....................................................................6

Supplementary Fig. 1 The morphology of curved perovskites films fabricated by spray-coating..........................................................................................................7

Supplementary Fig. 2 The simulation of spray-coating process.................................11

Supplementary Fig. 3 The relationship between the thickness of solid films and deposition time.......................................................................................................12

Supplementary Fig. 4 The solubility of the quasi-2D perovskites...............................13

Supplementary Fig. 5 The component characterization of the quasi-2D perovskite film fabricated by spray-coating. ...................................................................15

Supplementary Fig. 6 The spectra characterization of the quasi-2D perovskite film fabricated by spray-coating..........................................................................16

Supplementary Fig. 7 The narrow-band response characterization of the quasi-2D perovskite film fabricated by spray-coating. .....................................................18

Supplementary Fig. 8 The performance of the planar narrow-band photodetector...21

Supplementary Fig. 9 The performance of the hemispherical narrow-band photodetector.........................................................................................................23

Supplementary Fig. 10 The wide-angle detection of the hemispherical photodetector. .......................................................................................................................25

Supplementary Fig. 11 The resolution of the hemispherical photodetector. .............26

Supplementary Fig. 12 The micro-array imaging of the hemispherical photodetector .......................................................................................................................28

**Supplementary References** ......................................................................................29

## Supplementary Notes

**Materials:** All chemicals were used without further purification. Lead (II) iodide ($PbI_2$, 99.99% powder), lead (II) bromide ($PbBr_2$, 99.99% powder), 2,9-dimethyl-4,7-diphenyl-1,10-Phenanthroline (BCP, >99% HPLC), Poly[bis(4-phenyl) (2,4,6-trimethylphenyl) amine (PTAA, Mw: 1000-10000 by GPC), Poly(3,4-ethylenedioxythiophene)-poly(styrenesulfonate) dry re-dispersible pellets (PEDOT: PSS, Clevious$^{TM}$ HTL Solar 1-1.2 wt% solution in water and PH1000) were purchased from Xi'an p-OLED. N, N-Dimethylformamide anhydrous (DMF, 99.9%) and dimethyl sulfoxide anhydrous (DMSO, ≥99.7%), acetonitrile anhydrous (ACN, 99.9%) were purchased from Energy Chemical. L-ascorbic acid (L-AA, 99%), $C_{60}$ (99.5%), Sigma Aldrich. $SnO_2$ (15%$_{wt}$ aq.) were purchased from Alfa.

Formamidine hydroiodide (FAI, 99%), formamidine hydrobromide (FABr, 99%) phenethylamine hydroiodide (PEAI, 99%), phenethylamine hydrobromide (PEABr, 99%), cesium iodide (CsI, ≥99.9%), were purchased from Great Cell Solar.

**General Information:** EQE spectra were recorded under illumination of monochromatic light from the xenon lamp using a monochromator (Zolix) and detected by a computer-controlled lock-in amplifer. UV-visible absorption spectra were obtained using a Shimadzu 3600 UV-visible-NIR spectrophotometer. Photoluminescence spectra were obtained using Omni-λ3007i with a 375 nm laser. XRD data were collected using a PANalytical B.V.-Empyream Diffractometer with Cu Kα radiation. The SEM cross-section images were

acquired by Hitachi Cold Field Emission SEM with Bruker X-ray Photoelectron Spectrometer. High resolution ultraviolet photoemission spectro (UPS) were measured in an integrated ultrahigh vacuum system equipped with multitechnique surface analysis system (VG ESCALAB MK II spectrometer).

## Supplymentary Methods

### The simulation of the flow field distribution

ANSYS education edition 2022a was used to simulate the flow field distribution. The liquid phase is acetonitrile, and the gas phase is air. The initial pressure of the inlet is 170000 Pa. The flow velocity is $1.5 \times 10^{-6}$ $kg \cdot s^{-1}$. The time step is 0.01s. The liquid phase nozzle diameter is 0.2 mm, and the gas phase nozzle is toroidal with 0.3 mm of inner diameter and 0.4 mm of external diameter.

### Imaging system.

The imaging system is a home made system composed of X-Y displacement of the machine, detector, object, LEDs, SR570 pre-amplifier, SR830 lock-in amplifier, and RIGOL DG1022U function generator. The LEDs (3W) with different wavelengths were fixed on the displacement of a machine and were driven. The length of every step is 500 μm, and the moving range is 5×5 cm or 6×6 cm. The frequency of the light source is 70 Hz. The array imaging system is composed of a detector, object, Series 2400 SourceMeter, and LEDs.

### The crystallization time extracted from microscope.

The glass (1.5 cm×1.5 cm) was treated with UV-$O_3$ for 20 min and placed on a hot plate before being heated to 100 °C. Five μL perovskite precursor was dropped onto the glass. The concentration of perovskite is the same as that of the mixed quasi-2D perovskite ($FAPbI_3$, 0.45 M, w 30%$_{mol}$ MACl; $PEA_2PbI_4$, 0.15 M, w 30%$_{mol}$, MACl; $PEA_2FA_3Pb_4I_{13}$, w 30%$_{mol}$ MACl). The crystallization process was recorded by a microscope. During crystalization, the liquid and solid film’s grey levels are different. Thus, the grey level of different pictures captured from the microscope is continuously varies until the solid film formed. We statistics the change of grey levels during film formation and gave a curve to show this process. The microscope videos of the process of crystallization were converted to grayscale images. The frame rate of the videos is 30 Hz. The pixels were collected if the grayscale was greater than 12.75 (This process was realized by homemade script). The time history of the ratio of collected pixels related to all pixels was plotted. The zero time of the process was the point of time when the droplet spread out completely.

## Supplementary Figures and Discussions

After spray-coating and thermal annealing, the perovskite ($PEA_2FA_3Pb_4I_{13}$) film with weak reflet finished crystallization onto the hemispherical substrate. The thick films can be polished by the polish paper like single-crystal to obtain smooth surfaces[1]. The smooth surface will contribute to the fabrication of devices with a more continuous electrode. Supplementary Fig. 1a shows optical photographs of perovskite films ($PEA_2FA_3Pb_4I_{13}$) with and without polishing. Supplementary Fig. 1b and Supplementary Fig. 1c show the SEM images of the polishing and without polishing surface on the curved film ($PEA_2FA_3Pb_4I_{13}$) surface. The cross profile of perovskite ($PEA_2FA_3Pb_4I_{13}$) films adhering to hemispherical substrates is shown in Supplementary Fig. 1d. The thickness of the film is ~ 22 μm.

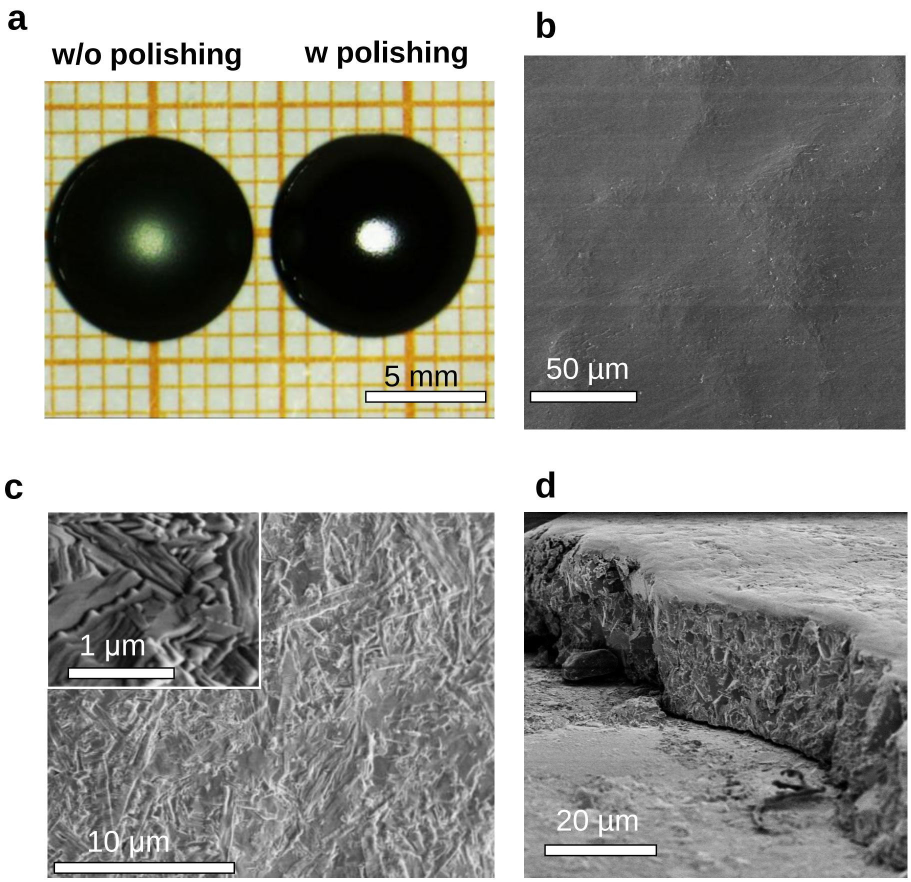


**Supplementary Fig. 1 | The morphology of curved perovskites films fabricated by spray-coating. a,** The optical photographs of perovskite ($PEA_2FA_3Pb_4I_{13}$) films fabricated on the hemispherical substrate through spray-coating methods. (Left: w/o polishing, Right: w polishing) **b,** The SEM image of the surface of perovskite ($PEA_2FA_3Pb_4I_{13}$) films deposited onto the hemispherical substrate fabricated by spray-coating method after mechanical polishing. **c,** The SEM image of the surface of perovskite ($PEA_2FA_3Pb_4I_{13}$) films deposited onto the hemispherical substrate fabricated by spray-coating method without polishing. (Inset: Locally magnified surface) **d,** The SEM image of the cross profile

of perovskite ($PEA_2FA_3Pb_4I_{13}$) films deposited onto the hemispherical substrate fabricated by spray-coating methods after mechanical polishing.

To better model the spray-coating process, we analyzed the relationships between the thickness of the liquid film and the solid (perovskite) film. The profile of liquid film is shown in Supplementary Fig. 2a. The cutting line is the transverse centerline of the liquid film shown in the inset. The edges of the liquid film were fitted by the Gauss curve (violet line). The film between the two Gauss curves' peaks was considered uniform with a distance of 3.75 cm.

Similarly, the profile of liquid thickness whose cutting line is along the longitudinal centerline of the liquid film was also analyzed in Supplementary Fig. 2b. The green line is the fitting curve, the FWHMs of which is 0.68 cm. The shape of the liquid film was simplified as a uniform extruded body with a cross profile of Gauss curve. Supplementary Fig. 2c shows the superposition of two adjacent groups of liquid films. The distance between the center lines of these two liquid films is 0.8 cm. The thickness of peaks and valleys is 3.49 μm and 3.00 μm, respectively. After the layer-by-layer deposition, the ups and downs will be eliminated. The volume ratio of solid film and liquid film ($\frac{V_s}{V_l}$) can be calculated from the amount of solute by the following formula.

The volume ratio of solid film and liquid film ($\frac{V_s}{V_l}$) can be calculated from the amount of solute by the following formula (1).

$$\frac{V_s}{V_l} = \frac{n}{V_l} \cdot V_{crystal} \cdot N_A = c \cdot V_{crystal} \cdot N_A \quad (1)$$

where *n* and *c* are the amount of substance (mole) and mole concentration of perovskite respectively, $N_A$ is Avogadro constant, $V_{crystal}$ is the volume of the crystal lattice, $V_l$ and $V_s$ are the volume of liquid and solid film respectively. Here, the weighted average volume was adopted to the calculation of composite perovskites' $V_{crystal}$. For example, the weighted average volume of $PEA_2FA_{i-1}Pb_iI_{3i+1}$ ($V_{i,\,c}$) is shown in the following formula (2).

$$V_{i,c} = \frac{1}{i} V_{PEA_2PbI_4} + \frac{i-1}{i} V_{FAPbI_3} \quad (2)$$

where *i* is the n value of quasi-2D perovskites, $V_{PEA_2PbI_4}$ is the volume of the crytal lattice of $PEA_2PbI_4$ (624.58 Å$^3$, Z value (Z) = 4), $V_{FAPbI_3}$ is the volume of the crytal lattice of $FAPbI_3$ (256.44 Å$^3$) [2,3]. When it comes to *i* = 4, the $V_{4,\,c}$ is 348.48 Å$^3$. The concentration of perovskite ($PEA_2FA_3Pb_4I_{13}$) is 0.6 M (The concentration of $Pb^{2+}$ is 0.6 M). The volume ratio of solid film and liquid film ($\frac{V_s}{V_l}$) is 0.13. The relationship (3) of the solid film and liquid film is shown in Supplementary Fig. 2d.

$$\frac{V_l}{V_s} = \frac{H_l}{H_s} \quad (3)$$

Supplementary Fig. 2e shows the simulation result of the spray-coated film with an area of 12 cm$^2$ (3.75 cm×3.20 cm), and the film thickness is around 0.43 μm. The effective area of the film is shown in Supplementary Fig. 2f. The length ($L_l$) of the area is 3.75 cm, as Supplementary Fig. 2a calculated, and the width ($W_l$) of the area is 0.8 cm with three spray

tracks. The average thickness is 3.39 μm ($H_l$). The average thickness of solid film ($H_s$) is 0.44 μm at the condition of Supplementary Fig. 2f. The deposition volecity of solid film ($V_{s,\,t}$) can be calculated by following formula (4).

$$V_{s,t} = \frac{(m-1)\times W_l \times L_l \times H_s}{\Delta t \times m} \tag{4}$$

where $\Delta t$ is the deposition time for each track (1 s), $m$ is the number of tracks ($m \geqslant 2$). In the condition of m → ∞, thus, the deposition volecity of solid film is $1.28 \times 10^{-4}$ $cm^3 \cdot s^{-1}$. The solid thickness ($H_{s,}$ $(S, t)$) given area and time was obtained in equation (5). The thickness of the different deposition time was collected and shown in Supplementary Fig. 3a (S = 25 $cm^2$). When $t$ = 900 s, $S$ = 36 $cm^2$, thickness of film is 32.00 μm (Supplementary Fig. 3b).

$$H_s(S,t) = \frac{V_{s,t} \times t}{S} \tag{5}$$

where $S$ is the area to deposition the films, $t$ is the deposition time.

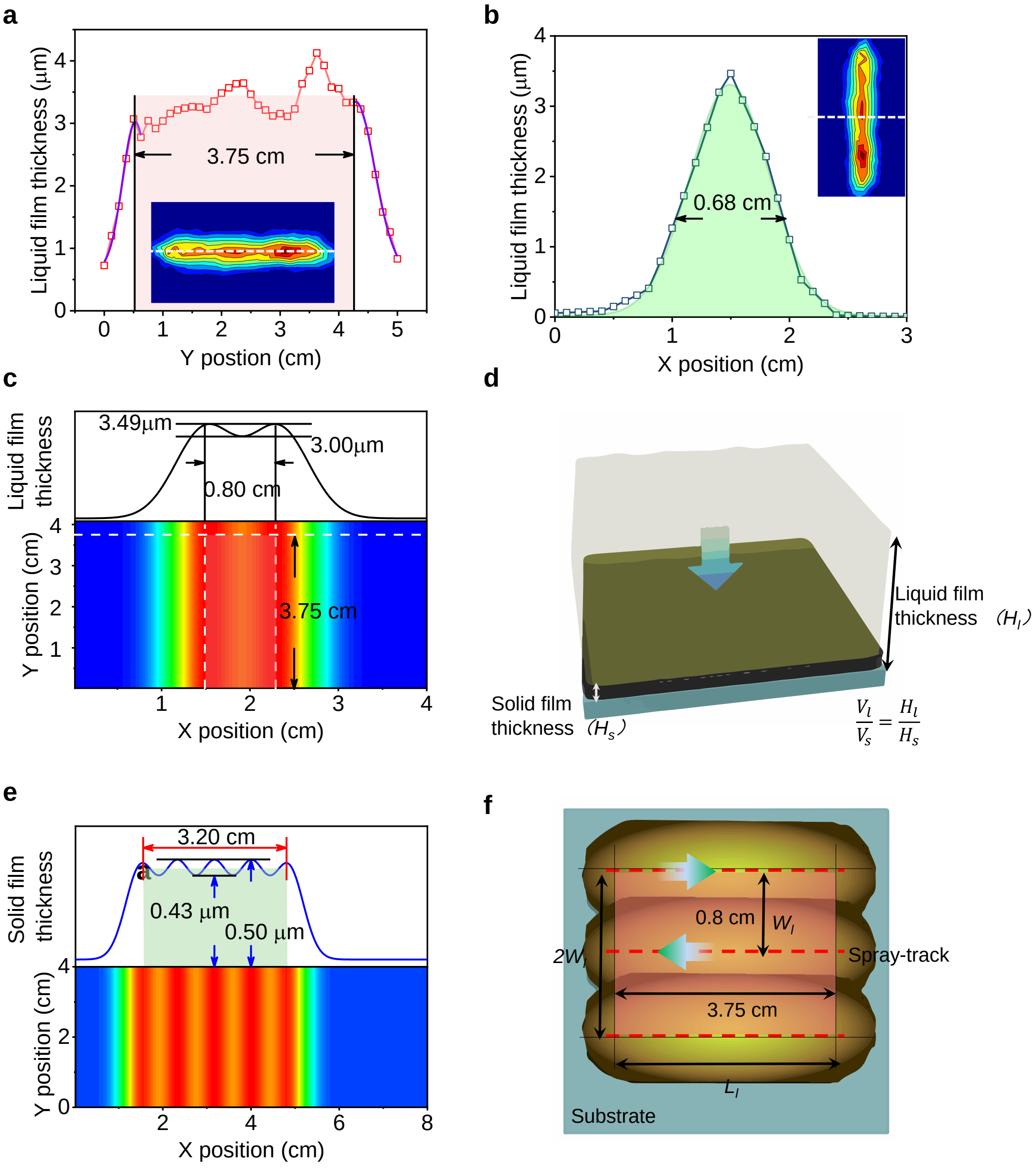


**Supplementary Fig. 2 | The simulation of spray-coating process. a,** The profile of liquid film calculated by numerical simulation. The profile position is along the transverse center line of the liquid film (refer to inset). **b,** The profile of liquid film calculated by numerical simulation. The profile position is along the longitudinal center line of the liquid film (refer to inset). **c,** Method for determining spray track. The profile of liquid film was fitted by

Gauss curve and the curve was shifted by a distance of 0.8 cm (~ 1.2 times of FWHMs) along x axis. **d,** The schematic diagram of thickness relationship of liquid and solid films. **e,** The peak superposition from five adjacent peaks of solid film. The solid film thickness is calculated by the simulated result of liquid film thickness. **f,** The schematic diagram of spray-track and the effective area of films fabricated by spray-coating.

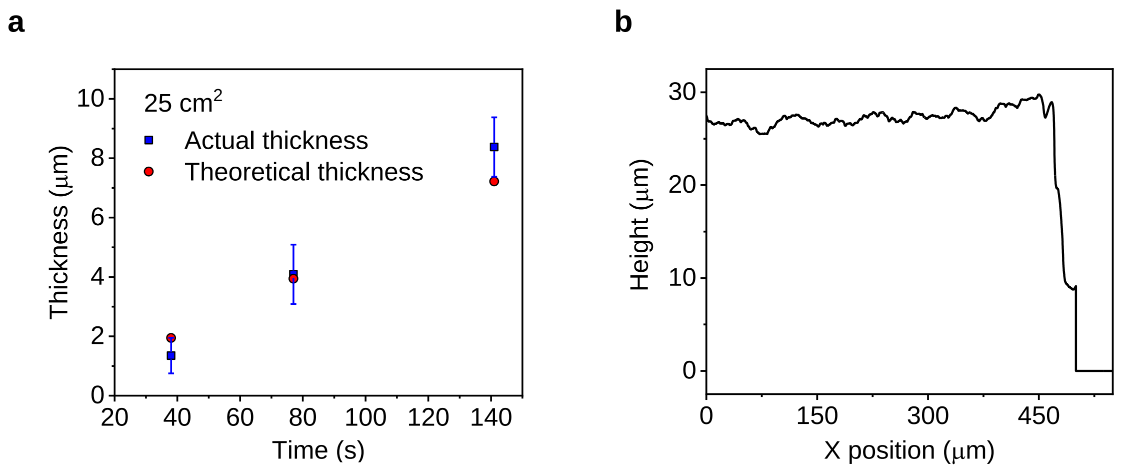


**Supplementary Fig. 3 | The relationship between the thickness of solid films and deposition time. a,** The actual and theoretical thickness at different deposition time on a 25 $cm^2$ substrate. The error bar is the range of film thickness. **b,** Actual thickness of solid film fabricated by spray-coating onto the 36 $cm^2$ substrate.

Supplementary Fig. 4 shows the solubility of 2D and 3D perovsktes. The solubility of 2D perovskites is higher than 3D perovskites at high temperatures in mixed solvent acetonitrile (ACN)/ N, N-dimethylformamide (DMF) = 1/1 (v/v). The solubility was

measured by preparing the supersaturated solution of perovskites at different temperatures. The solvent was removed from the supernatant liquor after being weighed. The solute was collected and weighed. The density of mixed solvent was measured at 0.84 g $cm^{-3}$ at room temperature, which we considered a constant value.

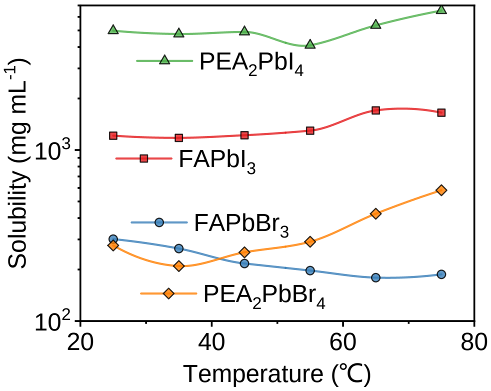


**Supplementary Fig. 4 | The solubility of the quasi-2D perovskites.** The solubility at different temperature of different perovskites ($PEA_2PbI_4$, $FAPbI_3$, $PEA_2PbBr_4$, and $FAPbBr_3$) in mixed solvent acetonitrile (ACN)/ N, N-dimethylformamide (DMF) = 1/1 (v/v).

To characterize the perovskite ($PEA_2FA_3Pb_4I_{13}$) films fabricated by spray-coating, the XRD spectra were measured. Methylammonium chloride (MACl) is an effective additive to assist $FAPbI_3$ forming α-phase. In fact, pure $PEA_2FA_3Pb_4I_{13}$ (w/o MACl) can also form

the black phase shown in Supplementary Fig. 5a. The addition of MACl can also include influent the crystal behavior in spray-coating case, which have been reported in spin-coating and balding coating cases. The main peaks (13.7° and 28.0°) of $PEA_2FA_3Pb_4I_{13}$ were identified refer to the 3D perovskite ($FAPbI_3$), which reflects the bond length of Pb-I (0.32 nm). It can also be identified refer to the quasi-2D and 2D perovskite, where the indices of crystal face (*hkl*) is (111) and (222). This difference comes from the difference in the cell definition, although the single-crystal data of $FA^+$ based quasi-2D perovskite have not been reported. The peak identification of the film without adding MACl can be analyzed qualitatively. The $FA^+$ based quasi-2D perovskite can be considered approximately tetragonal crystal system, where $a = b = d_{100,\,010} = 0.87$ nm $c = d_{001} = 1.61 + (n - 1)\times 0.63$, *a*, *b*, and *c* are the lattice parameters, *d* is the distance between lattice plane, n is the n value of quasi-2D perovskites. The value of *d*, the distance between adjacent planes in the set (*hkl*), calculated by following equation (6).

$$\frac{1}{d^2} = \frac{h^2+k^2}{a^2} + \frac{l^2}{c^2} \quad (6)$$

Detecting the species in materials through transient absorption (TA) spectra (Supplementary Fig. 5b) is an effective method to characterize the quasi-2D perovskites widely reported [4,5] .Almost no quasi-2D specie was found in the film of perovskite ($PEA_2FA_3Pb_4I_{13}$) fabricated by spray-coating, which means the 2D perovskite and 3D perovskite existed in a separated phase.

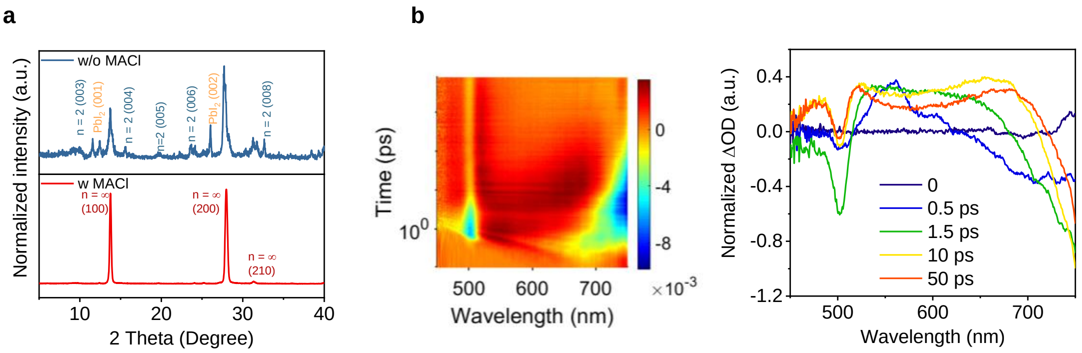


**Supplementary Fig. 5 | The component characterization of the quasi-2D perovskite film fabricated by spray-coating. a,** The XRD spectra of perovskite ($PEA_2FA_3Pb_4I_{13}$) films (w and w/o MACl) fabricated by spray-coating. **b,** The transient absorption (TA) spectra of perovskite ($PEA_2FA_3Pb_4I_{13}$) films (w MACl) fabricated by spray-coating.

The UPS of $PEA_2FA_3Pb_4I_{13}$ was measured and exhibited an energy-level structure (Fig. 6a), evidence that $PEA_2FA_3Pb_4I_{13}$ film fabricated by spray-coating is more similar to the 3D perovskite compared to 2D perovskite. The absorption coefficient and normalized PL spectra of $PEA_2FA_3Pb_4I_{13}$ were shown in Supplementary Fig. 6b, which means the spectral behavior of $PEA_2FA_3Pb_4I_{13}$ films is closer to 3D perovskite ($FAPbI_3$) not influent the response wavelength of photodetectors.

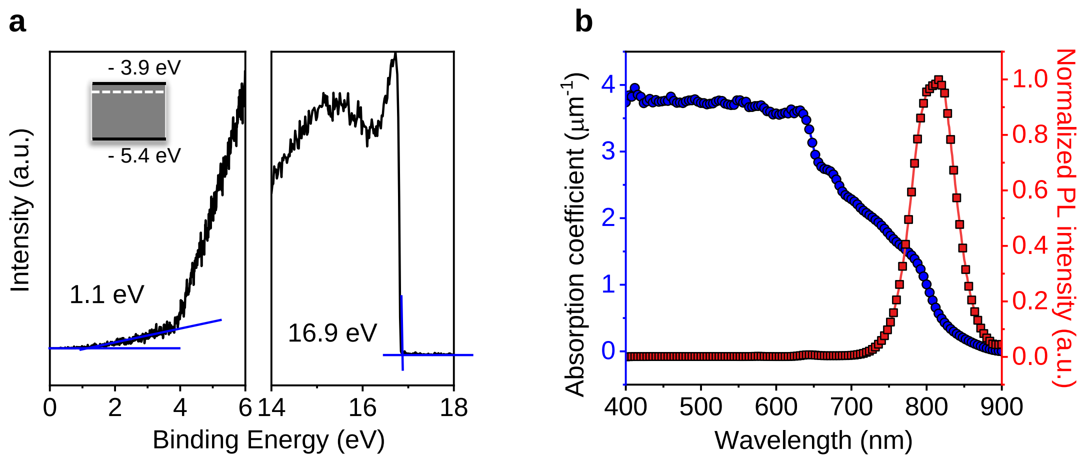


**Supplementary Fig. 6 | The spectra characterization of the quasi-2D perovskite film fabricated by spray-coating. a,** The UPS spectra and energy-level structure of perovskite ($PEA_2FA_3Pb_4I_{13}$) film fabricated by spray-coating. **b,** The absorption coefficient and PL intensity of the perovskite ($PEA_2FA_3Pb_4I_{13}$) film fabricated by spray-coating.

Supplementary Fig. 7a shows the device's cross profile (ITO/perovskite/Au) with a thickness of ~ 10 μm with good crystallization, which is enough to recombine the carriers from short-wavelength as Fig. 7b and Fig. 6c show, which are the normalized charge carriers' density calculated from absorbance spectra and μτ product at different distance and wavelength. The electron mobility ($\mu_e$) of the film ($PEA_2FA_3Pb_4I_{13}$) fabricated by spray-coating was evaluated in Supplementary Fig. 7d, which is $6.53 \times 10^{-5}$ $cm^2{\cdot}V^{-1}{\cdot}s^{-1}$ close to the mobility of quasi-2D perovskites. The electron mobility can be obtained by the equation (7).

$$\mu = \frac{d^2}{V \times t} \tag{7}$$

where $\mu$ is the charge carriers' mobility, $d$ is the thickness of perovskite film, $V$ is the applied voltage, and $t$ is the transit time of the charge carriers. The wavelength of the exciting light is 337 nm, where the carriers are generated at the surface of the film. We evaluated the short wavelength cut-off ratio of the device (ITO/perovskites/Au) by comparing the EQE ratio between 820 nm (response wavelength) and 700 nm (shielding wavelength). Fig. 7e and Fig. 6f show the influent of 2D components and the thickness of films. This self-filtering capacity is outstanding compared to other perovskites longitudinal film devices.

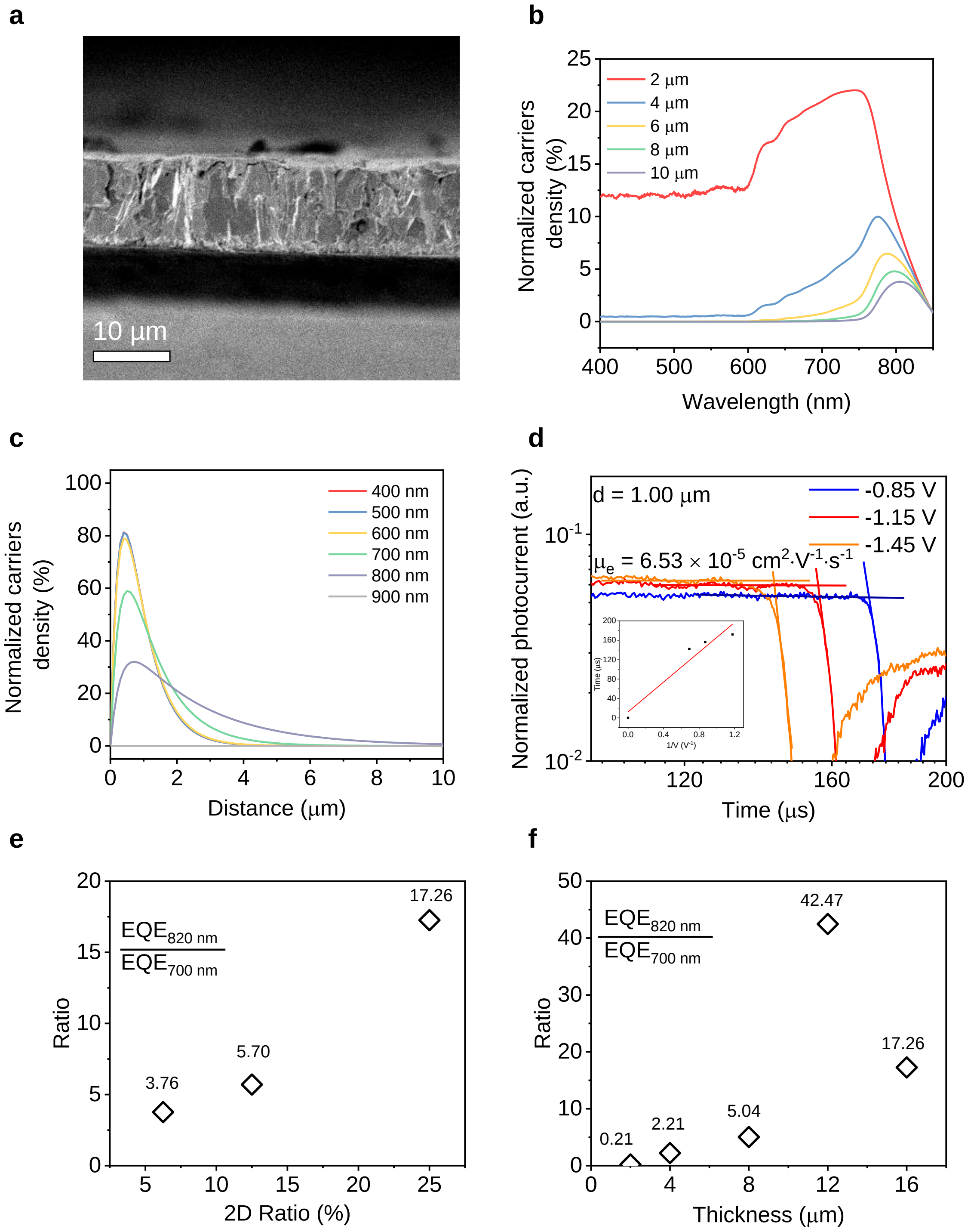


**Supplementary Fig. 7 | The narrow-band response characterization of the quasi-2D perovskite film fabricated by spray-coating. a,** The SEM image of a device's cross profile (ITO/perovskite/Au). **b,** The normalized charge carriers' density of perovskite

($PEA_2FA_3Pb_4I_{13}$) film fabricated by spray-coating at different wavelength. **c,** The normalized charge carriers' density of perovskite ($PEA_2FA_3Pb_4I_{13}$) film fabricated by spray-coating at different thickness. **d,** Normalized transient photocurrent curves of the photodetector under various bias. inset: the charge transit time verse the reciprocal of bias and the liner fit of the data **e,** The EQE ratio ($EQE_{820\ nm}/EQE_{700\ nm}$, 16 μm) to reflect the cut-off ratio of different devices (ITO/perovskites/Au). The perovskites were mixed with different ratios of $PEA_2PbI_4$ and $FAPbI_3$. **f,** The EQE ratio ($EQE_{820\ nm}/EQE_{700\ nm}$, 16 μm) to reflect the cut-off ratio of different devices (ITO/perovskites/Au) with different thickness of the photosensitive layer.

Then we designed a planar narrow-band photodetector with the structure of ITO/PEDOT: PSS/perovskites/$C_{60}$/BCP/Au (Supplementary Fig. 8a). The EQE of this photodetector is 5.3% at -0.6 bias (Supplementary Fig. 8b), with an acceptable cut-off ratio of 20.35, which is advanced compared to the self-filtering narrow-band photodetectors.[6,7] The responsivity (R) of 0.035 $A{\cdot}W^{-1}$ at 811 nm and –0.6 V bias was calculated from EQE data and shown in Supplementary Fig. 8c. Following is the formula (8) to obtain the responsivity.

$$R = \frac{EQE \times q}{hv} \tag{8}$$

where $q$ is the absolute value of electron charge, $h\nu$ is the energy of one photon at the corresponding wavelength. Response time was evaluated in Fig. 8d with $t_{up}$ = 1.16 ms and $t_{fall}$ = 0.40 ms, which is limited to the thickness of film, and the mobility of carriers and the charge and discharge of the recombination center. The current density (J)-voltage (V) curves at different light flux intensities are shown in Supplementary Fig. 8e. The specific detectivity ($D^*$) of the planar photodetector was approximately calculated by the following formula (9) of ~$10^{11}$ Jones at response wavelength, where the shot noise is assumed to be the dominant contribution (Supplementary Fig. 8f).

$$D^* \approx \frac{R}{(2\times q\times J_d)^{0.5}} \tag{9}$$

where $J_d$ is the dark current density.

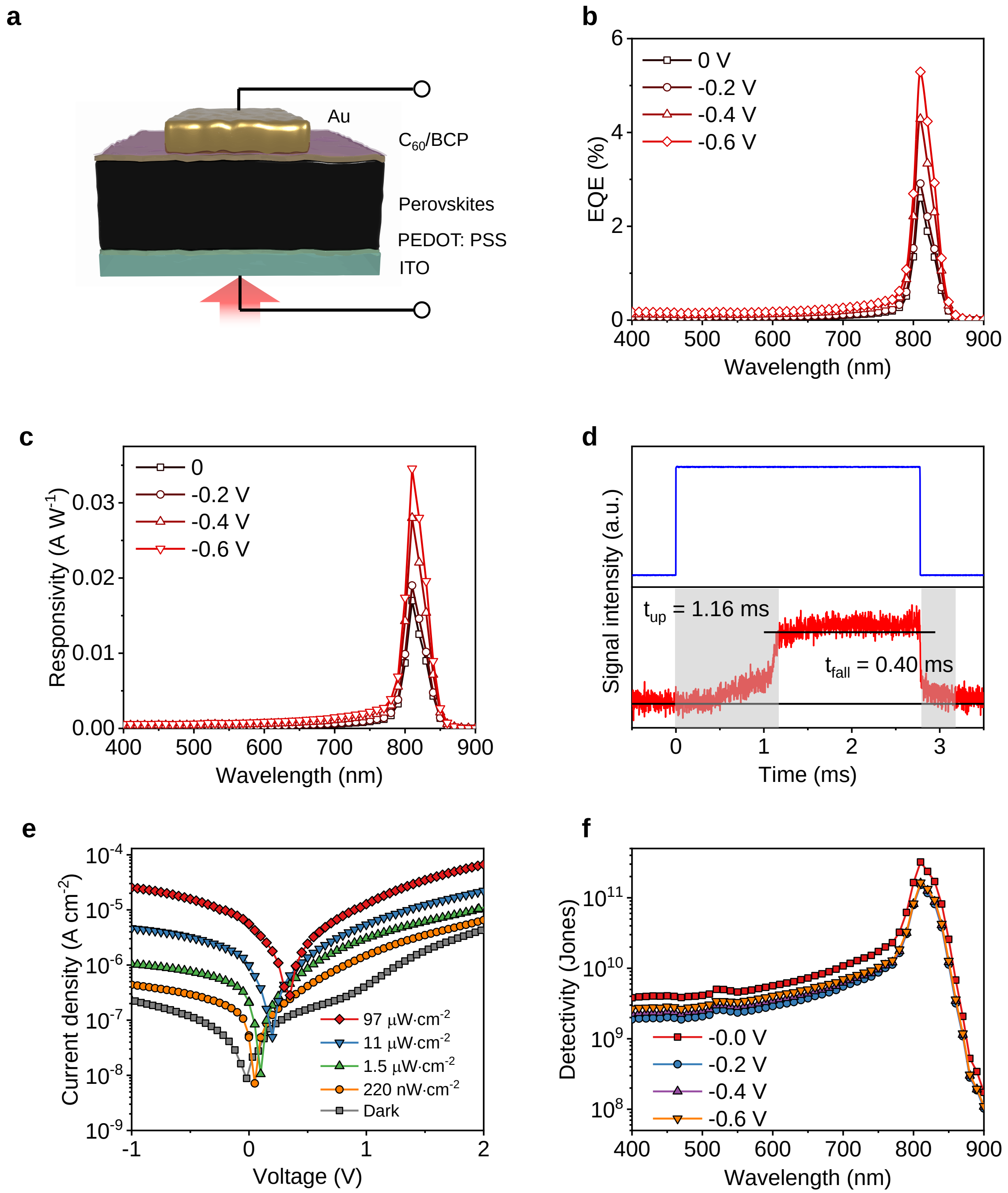


**Supplementary Fig. 8 | The proformance of the planar narrow-band photodetector.**

**a,** The device structure (ITO/PEDOT: PSS/perovskites/$C_{60}$/BCP/Au) of planar photodetectors (Active area: 0.1 $cm^2$). **b,** The EQE spectra under different bias of the planar photodetectors. **c,** The responsivity under different bias of the planar photodetectors. **d,**

The response time of the planar photodetectors (0 V). **e,** The photocurrent density under different irradiation intensity at 820 nm and dark current density of the planar photodetector. **f,** The detectivity under different bias of the planar photodetectors.

The hemispherical device was partially stripped and studied by SEM. The thickness of the film is uniform (Supplementary Fig. 9a). The SEM image of the cross profile of the hemispherical photodetector fabricated by spray-coating methods is shown in Supplementary Fig. 9b. The J-V curves of the hemispherical photodetector at different light flux intensities are shown in Supplementary Fig. 9c. The performance of the hemispherical photodetector is comparable to the planar one. The EQE of hemispherical photodetectors is shown in Supplementary Fig. 9d through tuning the I/Br ratio of the perovskites layer referred to the Supplementary Fig. 9e, which shows the relationship between the response wavelength of photodetectors and I/Br, with a simple linear correlation. The devices structure of photodetectors based on $PEA_2FA_3Pb_4I_5Br_8$, $PEA_2FA_3Pb_4I_2Br_{11}$, and $PEA_2FA_3Pb_4Br_{13}$ is Cr/$SnO_2$/ $PEA_2FA_3Pb_4I_xBr_{13-x}$/$FAPbI_yBr_{3-y}$/PTAA/Cr, where x = 5, 2, 0; y = 1.15, 0.46, 0, which is a n-i-p device structure. The $FAPbI_yBr_{3-y}$ layer is the buffer layer, which can optimize the narrow-band response of the photodetector due to the increased recombination centers in the interface. Supplementary Fig. 9f shows the shelf stability and operational stability of the photodetector at R.H. 20%~60%. The

hemispherical device has connected to a resistance of 50 Ω and placed under 0.25 sunlight without any encapsulation to measure the operational stability.

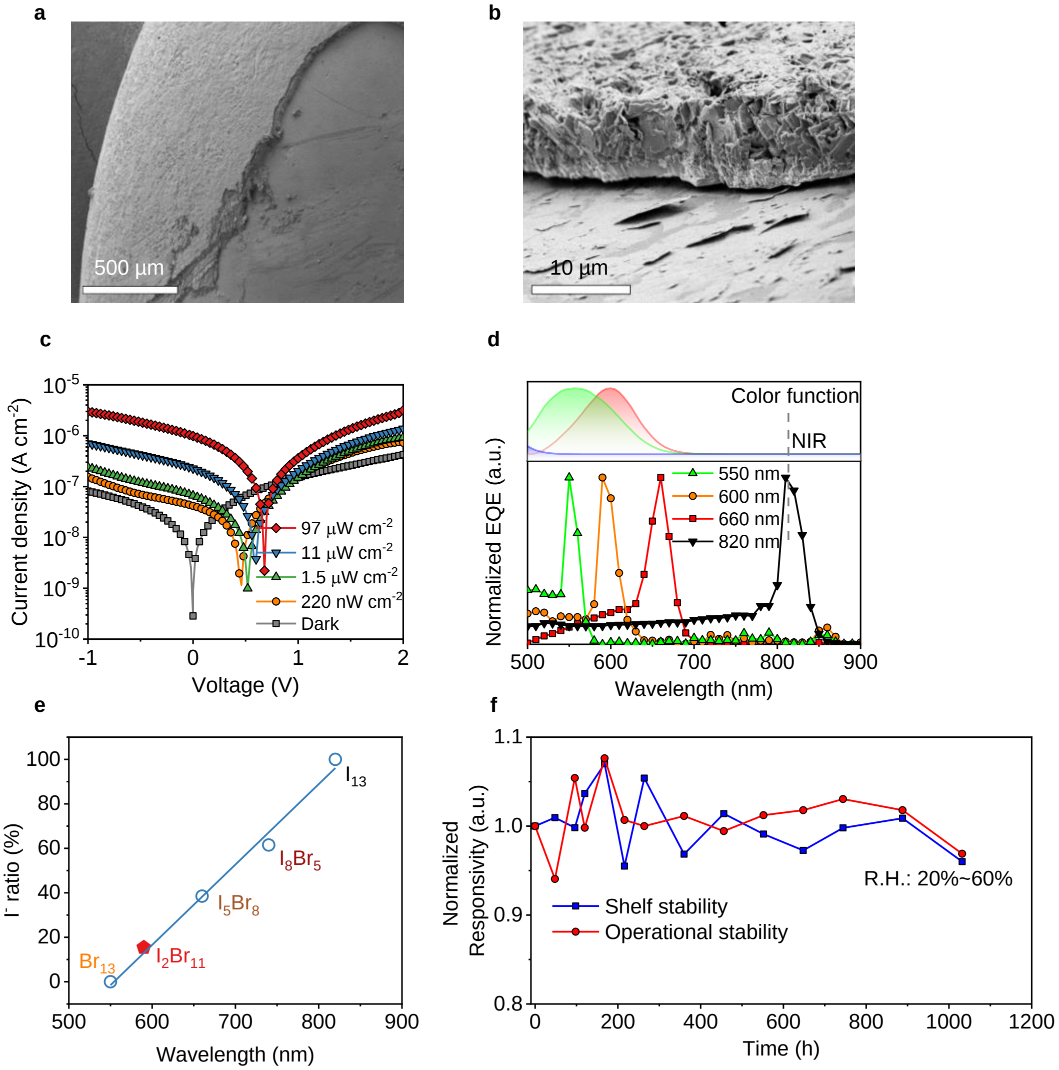


**Supplementary Fig. 9 | The performance of the hemispherical narrow-band photodetector. a,** The SEM of the surface of the hemispherical device partially stripped. **b,** The SEM image of the cross profile of the hemispherical photodetector. **c,** The photocurrent density under different irradiation intensity at 820 nm and dark current

density of the hemispherical photodetector. **d,** The EQE spectra of hemispherical photodetectors with different I/Br ratio (820 nm $PEA_2FA_3Pb_4I_{13}$, 660 nm $PEA_2FA_3Pb_4I_5Br_8$, 600 nm $PEA_2FA_3Pb_4I_2Br_{11}$, and 550 nm $PEA_2FA_3Pb_4Br_{13}$) at 0 V bias. **e,** The response wavelength of narrow-band hemispherical photodetectors through controlling the I/Br ratio of perovksites ($PEA_2FA_3Pb_4I_{13}$, $PEA_2FA_3Pb_4I_8Br_5$, $PEA_2FA_3Pb_4I_5Br_8$, $PEA_2FA_3PbI_2Br_{11}$, and $PEA_2FA_3Pb_4Br_{13}$), **f**, The stability of hemispherical photodetectors.

The theoretical transmittance (*T*) on planar perovskite surface was calculated by the transformation of Fresnel formula (10). The average of the intensity of two orthogonal polarized light ($T_{\parallel}$), ($T_{\perp}$) was considered as the the theoretical transmittance (*T*).

$$T = \frac{n_2}{2n_1}\left|\frac{2n_1\cos i_1}{n_1\cos i_1 + n_2\cos i_2}\right|^2 + \frac{n_1}{2n_2}\left|\frac{2n_1\cos i_1}{n_1\cos i_2 + n_2\cos i_1}\right|^2 \quad (10)$$

where $n_1$ and $n_2$ are refractive index of air and perovskite, respectively, $i_1$ is the angle of incidence, $i_2$ is the angle of refraction. Here, we set $n_1 = 1$ (air), $n_2 = 2$ (perovskite). The $n_2$ was used the approximate value from ellipsometer. The schematic diagram of the imaging system is shown in Supplementary Fig. 10b with the starting angle ($\vartheta_1$) and the end angle ($\vartheta_2$) calculation equation (11)

$$\vartheta_j = tan^{-1}\frac{h}{l_j} \quad (j = 1, 2) \quad (11)$$

where $l_j$ ($j$ = 1, 2) is the distance between the edge of the object and the normal line of the photodetector, and $h$ is the distance between the plane of the photodetector and that of the object. The photodetector is rotatable, and the object is removable, which can adjust the angle of incident light. When the photodetector was rotated, the $\vartheta_j$ should be calibrated in following equation (12).

$$\vartheta_j = tan^{-1}\frac{h}{l_j} - \xi \quad (j = 1, 2) \tag{12}$$

where $\xi$ is the angle of rotation. Clockwise is positive. Supplementary Fig. 10c shows the scheme of the $\vartheta_j$ when the $\xi$ is 90°. Supplementary Fig. 10d is the photocurrent of photodetectors under area light source.

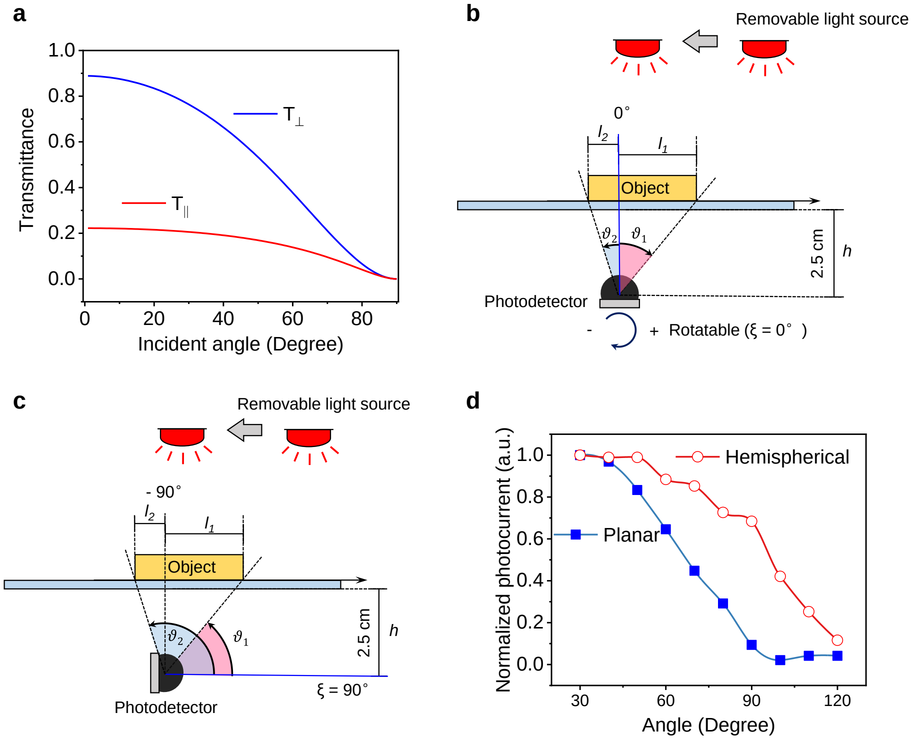


**Supplementary Fig. 10 | The wide-angle detection of the hemispherical photodetector.**

**a,** The relationship of incident angle and transmittance of light intensity based on Fresnel

formula at the planar surface of perovskite. **b,** Schematic diagram of the imaging system ($\xi$ = 0°). **c,** Schematic diagram of the imaging system ($\xi$ = 90°). **d,** The photocurrent of hemispherical and planar photodetectors under the different incident diffused light.

We calculated the resolution ratio of the large area narrow-band hemispherical photodetector from the image (Fig. 4d, -90° to -52°) through slanted-edge method for the modulation transfer function (MTF) calculation. The calculation details are mensioned in our previous publications. [8,9] We considered that the lower resolution comes from the large area of the photodetector with a bigger pixel.

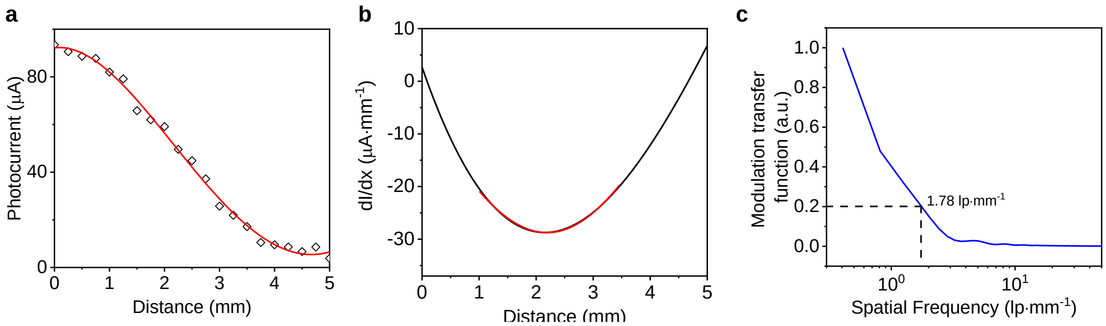


**Supplementary Fig. 11 | The resolution of the hemispherical photodetector. a,** Polynomial function fit for edge spread function (ESF(x)) **b,** The derived line spread function (LSF(x)) and Gauss fitted curve. **c,** The modulation transfer function (MTF) for the hemispherical photodetector.

Firstly, a white light LED was placed on the top of the system, and the photodetector was placed on the bottom of the system. The object was held by the glass and placed between the light source and the photodetector. Then, the light source was changed to the LED of 820 nm (invisible to the human eye) at the same position, which is the response wavelength of the perovskite ($PEA_2FA_3Pb_4I_{13}$). The photodetector is fixed. The photocurrent of different array points was measured by 2400 source meters. Matlab was used to restructure and smooth the photocurrent image. The raw image without smoothing is shown in Supplementary Fig. 12b. The PEDOT:PSS (PH1000) was used as electrodes in the array photodetector for better transmittance, and the transmittance of Cr and PEDOT:PSS (PH1000) were measured in Supplementary Fig. 12d.

a

| X/Y (A $cm^{-2}$) | 1 | 2 | 3 | 4 | 5 | 6 | 7 | 8 | 9 |
|---|---|---|---|---|---|---|---|---|---|
| 1 | 1.45E-07 | 9.98E-08 | 1.27E-07 | 1.20E-07 | 1.20E-07 | 1.26E-07 | 1.18E-07 | 1.19E-07 | 1.14E-07 |
| 2 | 1.42E-07 | 1.07E-07 | 3.67E-08 | 4.15E-08 | 4.17E-08 | 4.77E-08 | 4.64E-08 | 1.24E-07 | 1.29E-07 |
| 3 | 1.26E-07 | 1.13E-07 | 4.22E-08 | 1.08E-07 | 1.10E-07 | 1.15E-07 | 4.30E-08 | 9.21E-08 | 9.52E-08 |
| 4 | 1.22E-07 | 1.17E-07 | 5.08E-08 | 1.12E-07 | 1.38E-07 | 1.26E-07 | 4.25E-08 | 1.18E-07 | 1.32E-07 |
| 5 | 1.00E-07 | 1.09E-07 | 4.34E-08 | 1.09E-07 | 1.15E-07 | 1.20E-07 | 3.95E-08 | 1.06E-07 | 1.11E-07 |
| 6 | 1.41E-07 | 1.16E-07 | 4.43E-08 | 4.51E-08 | 3.87E-08 | 4.46E-08 | 4.01E-08 | 1.01E-07 | 1.01E-07 |
| 7 | 1.16E-07 | 1.15E-07 | 1.27E-07 | 1.24E-07 | 1.35E-07 | 1.30E-07 | 1.21E-07 | 1.16E-07 | 1.28E-07 |
| 8 | 1.33E-07 | 1.08E-07 | 1.38E-07 | 1.43E-07 | 1.35E-07 | 1.25E-07 | 1.39E-07 | 1.43E-07 | 1.41E-07 |
| 9 | 1.02E-07 | 1.14E-07 | 1.32E-07 | 1.11E-07 | 1.08E-07 | 1.12E-07 | 1.03E-07 | 1.22E-07 | 1.05E-07 |

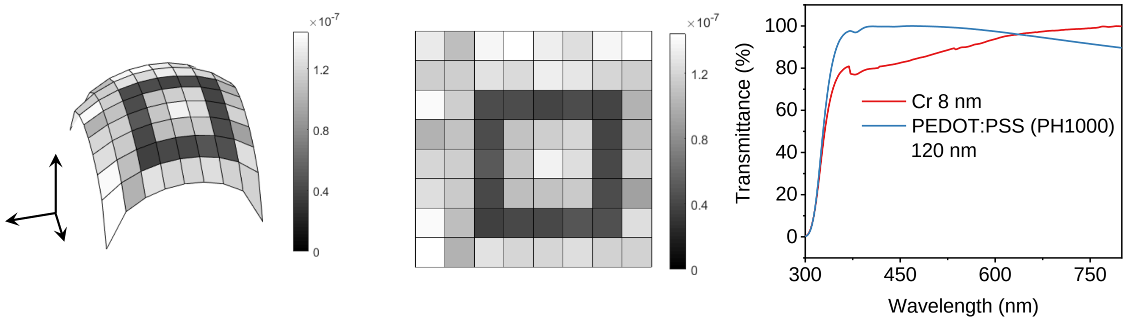


**Supplementary Fig. 12 | The micro-array imaging of the hemispherical photodetector.**

**a,** The photocurrent density signal of different arrays, where 1.45E-07 is $1.45 \times 10^{-7}$. **b, c,** The micro-array imaging result before smoothing. **d,** The transmittance of Cr (8 nm) and PEDOT:PSS (PH1000) (120 nm)

## Supplementary References


1 Ni, Z. *et al.* Resolving spatial and energetic distributions of trap states in metal halide perovskite solar cells. *Science* **367**, 1352-1358 (2020).

2 Wang, H. *et al.* Controllable $Cs_xFA_{1-x}PbI_3$ Single-Crystal Morphology via Rationally Regulating the Diffusion and Collision of Micelles toward High-Performance Photon Detectors. *ACS Appl. Mater. Interfaces* **11**, 13812-13821 (2019).

3 Du, K. Z. *et al.* Two-Dimensional Lead(II) Halide-Based Hybrid Perovskites Templated by Acene Alkylamines: Crystal Structures, Optical Properties, and Piezoelectricity. *Inorg. Chem.* **56**, 9291-9302 (2017).

4 Shao, M. *et al.* Over 21% Efficiency Stable 2D Perovskite Solar Cells. *Adv. Mater.*, e2107211 (2021).

5 Hu, J. *et al.* Synthetic control over orientational degeneracy of spacer cations enhances solar cell efficiency in two-dimensional perovskites. *Nat. Commun.* **10**, 1276 (2019).

6 Wang, J. *et al.* Self-Driven Perovskite Narrow-band Photodetectors with Tunable Spectral Responses. *Adv. Mater.* **33**, e2005557 (2021).

7 Fang, Y., Dong, Q., Shao, Y., Yuan, Y. & Huang, J. Highly narrow-band perovskite single-crystal photodetectors enabled by surface-charge recombination. *Nat. Photonics* **9**, 679-686 (2015).

8 Pan, W., Tan, M., He, Y., Wei, H. & Yang, B. Organic Amine-Bridged Quasi-2D Perovskite/PbS Colloidal Quantum Dots Composites for High-Gain Near-Infrared Photodetectors. *Nano Lett.* **22**, 2277-2284 (2022).

9 Liu, L. *et al.* Energy Transfer Assisted Fast X-ray Detection in Direct/Indirect Hybrid Perovskite Wafer. *Adv. Sci.*, e2103735 (2022).
